# Tamoxifen and bone morphogenic protein-7 modulate fibrosis and inflammation in the peritoneal fibrosis model developed in uremic rats

**DOI:** 10.1186/s10020-019-0110-5

**Published:** 2019-08-28

**Authors:** Filipe M. O. Silva, Elerson C. Costalonga, Cleonice Silva, Ana C. O. Carreira, Samirah A. Gomes, Mari C. Sogayar, Camilla Fanelli, Irene L. Noronha

**Affiliations:** 1 Laboratory of Cellular, Genetic, and Molecular Nephrology, Renal Division, University of São Paulo Medical School, Av. Dr. Arnaldo, 455, 4o andar, Lab 4304, São Paulo, CEP 01246-903 Brazil; 2 Cell and Molecular Therapy Center, University of São Paulo Medical School, São Paulo, Brazil; 3 Anatomy Department, University of São Paulo Veterinary and Zootecnology School, University of São Paulo, São Paulo, Brazil; 4 Biochemistry Department, Chemistry Institute, University of São Paulo, São Paulo, Brazil

**Keywords:** Peritoneal fibrosis, Peritoneal inflammation, Tamoxifen, BMP7, TGF-ß, Smads

## Abstract

**Background:**

Peritoneal fibrosis (PF) represents a long-term complication of peritoneal dialysis (PD), affecting peritoneal membrane (PM) integrity and function. Understanding the mechanisms underlying PF development in an uremic environment aiming alternative therapeutic strategies for treating this process is of great interest. The aim of this study was to analyze the effects of tamoxifen (TAM) and recombinant BMP7 (rBMP7) in an experimental model of PF developed in uremic rats.

**Methods:**

To mimic the clinical situation of patients on long-term PD, a combo model, characterized by the combination of PF and CKD with severe uremia, was developed in Wistar rats. PF was induced by intraperitoneal (IP) injections of chlorhexidine gluconate (CG), and CKD was induced by an adenine-rich diet. Uremia was confirmed by severe hypertension, increased blood urea nitrogen (BUN> 120 mg/dL) and serum creatinine levels (> 2 mg/dL). Uremic rats with PF were treated with TAM (10 mg/Kg by gavage) or BMP7 (30 μg/Kg, IP). Animals were followed up for 30 days.

**Results:**

CG administration in uremic rats induced a striking increase in PM thickness, neoangiogenesis, demonstrated by increased capillary density, and failure of ultrafiltration capacity. These morphological and functional changes were blocked by TAM or rBMP7 treatment. In parallel, TAM and rBMP7 significantly ameliorated the PM fibrotic response by reducing α-SMA, extracellular matrix proteins and TGF-ß expression. TAM or rBMP7 administration significantly inhibited peritoneal Smad3 expression in uremic rats with PF, prevented Smad3 phosphorylation, and induced a remarkable up-regulation of Smad7, an intracellular inhibitor of TGFβ/Smad signaling, contributing to a negative modulation of profibrotic genes. Both treatments were also effective in reducing local inflammation, possibly by upregulating IκB-α expression in the PM of uremic rats with PF. In vitro experiments using primary peritoneal fibroblasts activated by TGF-ß confirmed the capacity of TAM or rBMP7 in blocking inflammatory mediators, such as IL-1ß expression.

**Conclusions:**

In conclusion, these findings indicate important roles of TGF-ß/Smad signaling in PF aggravated by uremia, providing data regarding potential therapeutic approaches with TAM or rBMP7 to block this process.

**Electronic supplementary material:**

The online version of this article (10.1186/s10020-019-0110-5) contains supplementary material, which is available to authorized users.

## Introduction

Peritoneal dialysis (PD) is a well-established renal replacement therapy worldwide for the treatment of end-stage renal disease. However, continuous exposure to bioincompatible dialysis solutions, intermittent inflammation or infectious episodes can induce structural and functional changes of the peritoneal membrane (PM), which frequently lead to the failure of this treatment modality. The most important changes in the peritoneum observed in long term of PD includes progressive peritoneal fibrosis (PF) and neoangiogenesis, limiting the efficacy of solute transport, accounting for ultrafiltration loss (Williams et al. 2002; Lai and Leung 2010). In addition, systemic inflammation associated with the uremic state in patients with chronic kidney disease (CKD) can also compromise the PM, which directly contributes to the development of PF (Pecoits-Filho et al. 2002; Akchurin and Kaskel 2015).

Transforming growth factor-ß (TGF-ß) is considered a key molecule involved in progressive PF (Margetts et al. 2001; Loureiro et al. 2011). TGF-ß overexpression in the peritoneum induces extracellular matrix (ECM) proteins production and myofibroblast accumulation, leading to PM thickening (Margetts et al. 2001; Loureiro et al. 2011). In contrast, blocking TGF-ß in experimental PF induced by PD fluid exposure ameliorates peritoneal morphologic and functional changes (Loureiro et al. 2011).

TGF-ß/Smad signaling has been recognized as an important pathway in the development of PF (Lan 2011; Duan et al. 2014). Binding of active TGF-β to TGF-ß receptors present on cell-surface leads to downstream activation of Smad proteins. Phosphorylated (phospho)-Smad2 and Smad3 form heterodimeric complexes with Smad4, which translocate to the nucleus and regulate profibrotic gene transcription (Nakao et al. 1997). TGF-ß-mediated phosphorylation of Smad2/3 can be inhibited by Smad7, a well known intracellular antagonist of TGF-ß/Smad signaling (Nakao et al. 1997; Itoh and ten Dijke 2007).

Therapeutic approaches using molecules with anti-fibrotic properties that can interfere with TGF-ß/Smad signaling may represent interesting strategies for blocking PF development. One candidate is tamoxifen (TAM), an estrogen receptor (ER) modulator, effectively used for the treatment of patients with retroperitoneal fibrosis and encapsulating peritoneal sclerosis (Allaria et al. 1999; van Bommel et al. 2006). Tamoxifen has also been shown to inhibit renal fibrosis in a model of progressive CKD, by downregulating TGF-ß expression and decreasing the number of myofibroblasts in the renal interstitium (Dellê et al. 2012). Bone morphogenetic protein-7 (BMP7), which counteracts the biological functions of TGF-ß and exhibits anti-fibrotic properties, may represent another candidate, as demonstrated in different models of tissue fibrosis (Zeisberg et al. 2003; Sugimoto et al. 2007). Beneficial effects of tamoxifen and BMP7 on non-uremic animals exposed to PD fluid that developed PF have been reported so far (Loureiro et al. 2013; Loureiro et al. 2010).

Considering that most experimental PF studies have been established in animals with normal renal function, the induction of PF associated with uremia likely better translate the mechanisms involved in PM inflammation and fibrosis. In the present study, in order to more closely resemble the clinical settings, we established an experimental model of progressive PF in CKD animals with severe uremia.

Experimental PF can be induced using PD solutions through peritoneal catheters. Considering the complications related to long-term maintenance of catheters in rodents, accounting for catheter malfunction, infection, and high dropout rates, we chose a nonsurgical model, by exposing the PM to chlorhexidine gluconate (CG). CG injected intraperitoneally (IP) induces aseptic peritonitis with PM damage, leading to inflammation and tissue fibrosis (Suga et al. 1995; Hoff 2005). To avoid models that require surgery for CKD induction, as the 5/6 ablation model (Guo et al. 2007; Mortier et al. 2005; Zareie et al. 2005), in the present study CKD was accessed by subjecting the rats to an adenine rich diet (Yokozawa et al. 1986; Costalonga et al. 2017; Santana et al. 2013). This model is characterized by remarkable uremia and elevated levels of pro-inflammatory cytokines such as TNF-α, IL-1β and IL-6 (Santana et al. 2013).

The combination of these two pathological features, characterized by simultaneous PF and advanced CKD with severe uremia, provided a combo experimental model that reproduces the structural and functional scenario of long-term PD. The aim of the present study was to analyze and compare the effects of two antifibrotic strategies, namely tamoxifen and BMP7, in this clinical setting, to determine their usefulness as alternative therapeutic strategies, investigating the possible mechanisms underlying the development of local inflammation and PF in this process.

## Materials and methods

### Animal model and experimental groups

Sixty-eight adult male Wistar rats, weighting 250–300 g, were used in this study. All experimental procedures were approved by the Institutional Ethical Research Board (number 460/11). To induce CKD, rats received a 0.75% adenine-containing diet (Sigma Co, St. Louis, USA) for 30 consecutive days. PF was induced by IP injections of 0.1% CG for 15 consecutive days, after the establishment of kidney dysfunction. Treatments consisted of tamoxifen citrate (Nolvadex, Astra-Zeneca, Brazil) and purified rBMP7 (Bustos-Valenzuela et al. 2010).

Animals were divided into 6 groups. The **Control** group received standard diet. The **CKD Group** received adenine-rich diet for 30 days to induce CKD with severe uremia. The **PF Group** received normal diet and CG injections, IP, from day 15 until day 30, to induce PF. To mimic the clinical situation, the combo model was established, consisting of PF induction in CKD rats, denominated **PF/CKD Group**. The two groups with intervention therapy consisted of: **PF/CKD + TAM,** PF/CKD animals receiving tamoxifen citrate, 10 mg/Kg, daily, by gavage, from day 15 until day 30, and the **PF/CKD + rBMP7**, PF/CKD rats receiving rBMP7, 30 μg/Kg, IP, every 3 days, from day 15 until day 30.

At 0, 15 and 30 days, systolic blood pressure, serum creatinine and BUN levels were measured. On day 30, peritoneal function tests were performed, and then, animals were euthanized with 25–50 mg/Kg IP sodium pentobarbital. PM samples were collected from the left anterior abdominal wall.

### Peritoneal function test

For the peritoneal function tests, 0.09 ml/g body weight of 4.25% PD solution (Fresenius Medical Care, São Paulo, Brazil) was administered IP. Two hours later, the abdominal cavity was opened and the peritoneal fluid was drained, for UF measurements. UF values were calculated as the volume of fluid removed after 2 h minus the volume of fluid administered.

To evaluate the transport of small solutes across the peritoneum peritoneal fluid samples were centrifuged at 500 g for 5 min, and then, the glucose levels in the supernatant were measured (Cobas C111 Analyzer, Roche, Indianapolis, USA). The mass transfer of glucose through the peritoneum was calculated using the following formula: (initial dialysate glucose concentration X initial volume) – (final dialysate glucose concentration X final volume). The results obtained were corrected for animal weight.

### Peritoneal histology

PM samples were collected and fixed in buffered 10% formaldehyde solution. Sections of 2–3 μm thickness were stained with Masson Trichrome technique. Photographs of the entire length of the peritoneum (approximately 1 cm fragments) were taken at 200x magnification, and thickness was measured at three points in every single frame, using the Image ProPlus Software 7.0 (Media Cybernetics Inc., Bethesda, USA).

### Immunohistochemistry and immunofluorescence

Paraffin-embedded PM sections were incubated with the following antibodies: anti-rat ED1 (Serotec, Oxford, UK), anti-CD3 (Abcam, Cambridge, MA, USA), anti-rat α-smooth muscle actin (α-SMA) (Sigma), anti-PCNA (DAKO, Glostrup, Denmark), anti-phospho-Smad3 (Abcam, Cambridge, UK), and anti-IκB-α (Santa Cruz Biotechnology). An LSAB-AP System (DAKO) revealed with fast red dye (Sigma), and a NovolinkPolymer Detection System (Leica Microsystems, Newcastle, UK) revealed with diaminobenzidine were employed for antibody detection (Dellê et al. 2012; Santana et al. 2013).

The number of macrophages, T-cells, PCNA, phospho-Smad3, IκBα positive cells as well as the α-SMA staining area (%) was calculated relative to the whole peritoneal area, excluding anterior abdominal wall muscles, using ImagePro Plus 7.0 software (Media Cybernetics, Inc., Bethesda, USA).

Indirect immunofluorescence for Smad7 was carried out in paraffin PM sections, permeabilized with 0.2% Triton X-100. After incubation with anti-Smad7 (Abcam, Cambridge, UK) the samples were incubated with an anti-goat IgG FITC conjugated secondary antibody (Sigma). The sections were subsequently observed under immunofluorescence microscopy and quantified as a ratio of positive cells relative to the total number of cells present in the PM.

Quantitative assessment of angiogenesis was carried out by detection of capillary vessel density in paraffin PM sections using a DyLight 594-labelled Griffonia simplicifolia isolectin B4 (Vector Laboratories, CA, USA), which also detects newly formed blood vessels. Nuclei were counterstained with DAPI (Life). For each animal, at least ten microscopic fields were scored under 400x magnification using the 594 nm filter for isolectin and 540 nm for DAPI (Nikon Eclipse 80i microscope, Tokyo, Japan). The number of isolectin-B4 positive blood vessels in the PM were counted. The density of capillaries present in each slide was determined by the number of blood vessels divided by the area of PM, and expressed as the number of vessels/mm^2^.

### Quantitative real-time PCR (qRT-PCR) and detection of cytokines in the PM

Gene expression of ECM components, FSP-1, TGF-ß, VEGF, IL-1β, TNF-α and IL-6 cytokines, Smad3 and Smad7 was analyzed by quantitative real-time PCR (qRT-PCR) (Dellê et al. 2012). The following PCR cycle program was used: 10 min at 95 °C, followed by 40 cycles of 15 s at 95 °C for denaturation, 20 s at 60 °C for combined annealing, and 10s at 72 °C for extension. For detection of IL-1β, TNF-α and IL-6 cytokines at the protein level in the PM samples, a commercial MILLIPLEX® MAP kit (Millipore Corporation, Billerica, MA) was used.

### Cell culture experiments

Primary culture of fibroblasts obtained from Wistar rats peritoneum explants was performed. Briefly, pieces of 1 mm^3^ were seeded into 25cm^2^ flasks and cultured in DMEM/F12 (Gibco Corp, Carlsbad, CA, USA), supplemented with 20% FCS (Cultilab, Campinas, Brazil) and antibiotics (amphotericin, 2.5 mg/ml; ampicillin, 100 mg/ml; and streptomycin, 100 mg/ml; all from Gibco Corp), at 37 °C in a humidified atmosphere of 5% CO2. When cell outgrowth from the explants began, the remaining tissue was removed. After four passages, the cells displayed typical fibroblast morphology and were phenotypically characterized as fibroblasts by immunofluorescence, being positive for vimentin, and negative for α-SMA, desmin, and cytokeratin (Witowski and Jörres 2006).

The MTT assay (3-(4,5-dimethylthiazol-2yl)-2, 5-diphenyltetrazolium bromide, Life Technologies, Carlsbad, CA, EUA) was employed to assess cell viability and proliferation of peritoneal fibroblasts. Thereafter, 1 × 10^6^ peritoneal fibroblasts, grown in 24 well culture plates, were stimulated with TGF-β [26, 8 ng/mL] for 24 h and then incubated with tamoxifen citrate (5 μM, Sigma) or rBMP7 (200 ng/mL). After 24 h, cells were harvested and the expression of IL-1β, TNF-α and Smad7 was analyzed by qRT-PCR.

### Statistical analysis

Data are presented as the mean ± SEM, and all statistical analyses were performed using the Prism statistical program (GraphPad, San Diego, USA). One-way analysis of variance with pairwise comparisons according to the Newmann-Keuls formulation was used. *p* values equal to or lower than 0.05 were considered as significant.

## Results

### Experimental model of PF combined with CKD with uremia

CKD induced by adenine in rats promoted significant hypertension and marked increases in serum BUN and creatinine levels on day 15, reaching peak levels on day 30 (Table 1). PF was also successfully induced by local exposure of CG to PM. Despite the severity of this new experimental model, which combines PF with advanced uremia in rats, the overall mortality rate in the PF/CKD group was not different from the CKD group (Additional file 1: data 1).

**Table 1:** Comparative analysis of blood pressure, blood urea nitrogen (BUN) and serum creatinine levels in the different groups

	Blood Pressure (mmHg)			BUN (mg/dL)			Serum Creatinine (mg/dL)		
	Day 0	Day 15	Day 30	Day 0	Day 15	Day 30	Day 0	Day 15	Day 30
Control	120 ± 4	126 ± 4	123 ± 3	17 ± 2	24 ± 2	21 ± 3	0.37 ± 0.03	0.36 ± 0.06	0.3 ± 0.01
CKD	128 ± 2	162 ± 4^*^	175 ± 2^*^	15 ± 1	78 ± 6^*^	134 ± 12^*^	0.40 ± 0.04	0.70 ± 0.08^*^	1.81 ± 0.15^*^
PF	124 ± 1	123 ± 1^#^	135 ± 3^#^	18 ± 2	18 ± 1^#^	17 ± 1^#^	0.29 ± 0.04	0.33 ± 0.04^#^	0.37 ± 0.05^#^
PF/CKD	126 ± 3	180 ± 9^*†^	169 ± 4^*†^	16 ± 1	80 ± 10^*†^	124 ± 17^*†^	0.33 ± 0.02	1.05 ± 0.12^*#†^	2.12 ± 0.2^*†^
PF/CKD + TAM	128 ± 2	176 ± 5^*†^	166 ± 3^*†^	16 ± 2	59 ± 5^*†^	155 ± 12^*†^	0.32 ± 0.03	0.84 ± 0.1^*†^	1.72 ± 0.15^*†^
PF/CKD + rBMP7	129 ± 3	175 ± 5^*†^	180 ± 8^*†^	20 ± 1	82 ± 11^*†^	120 ± 9^*†^	0.38 ± 0.05	1.20 ± 0.15^*#†^	1.71 ± 0.12^*†^

Data are expressed as mean ± SEM

*p < 0.001 vs Control

^#^*p* < 0.01 vs CKD

^†^*p* < 0.01vs PF

### Tamoxifen and BMP7 protected against the development of PM fibrosis and neoangiogenesis

**Fig. 1:**
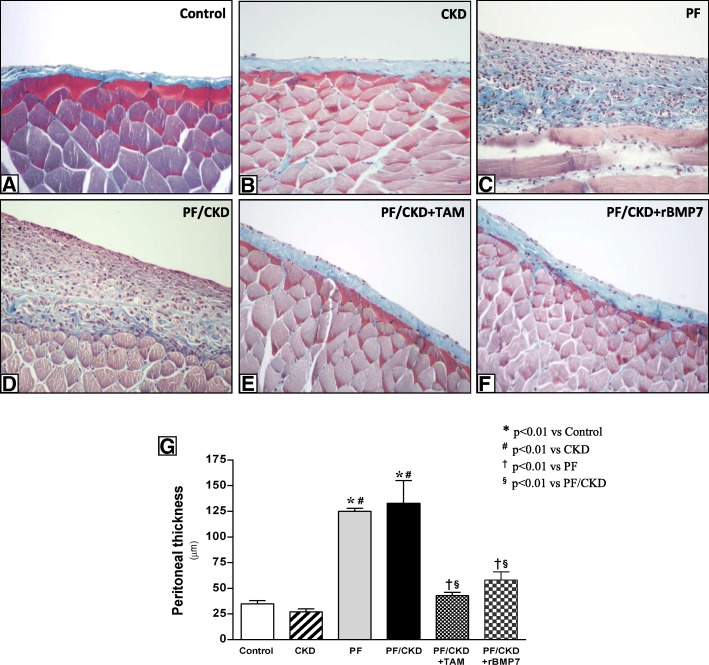
Histological features of peritoneal samples obtained from the different groups stained with Masson Trichrome (× 200). There were no morphological alterations in mesothelial, submesothelial or muscle cells in the Control group (**a**) or in animals with advanced CKD (**b**). CG induced marked thickening of the submesothelial peritoneal membrane, a change characterized by increased cellularity and collagen deposition, in the PF (**c**) and PF/CKD groups (**d**). Uremic rats with PF treated with TAM (**e**) or rBMP7 (**f**) exhibited preserved peritoneal membrane, as demonstrated via quantitative analysis (**g**). Overall ANOVA *p* < 0.0001. Post-test *p*-values: *p < 0.01 vs Control; ^#^*p* < 0.01 vs CKD, ^†^*p* < 0.01 vs PF, ^§^*p* < 0.01 vs PF/CKD

The establishment of PF in rats was confirmed by histological analyses showing severe membrane thickening in the PF and PF/CKD groups, four-fold greater than the Control and CKD groups (*p* < 0.01; (Fig. 1). Treatment with TAM or rBMP7 significantly ameliorated PM fibrosis and preserved PM morphology, similar to the Control and CKD groups.

**Fig. 2:**
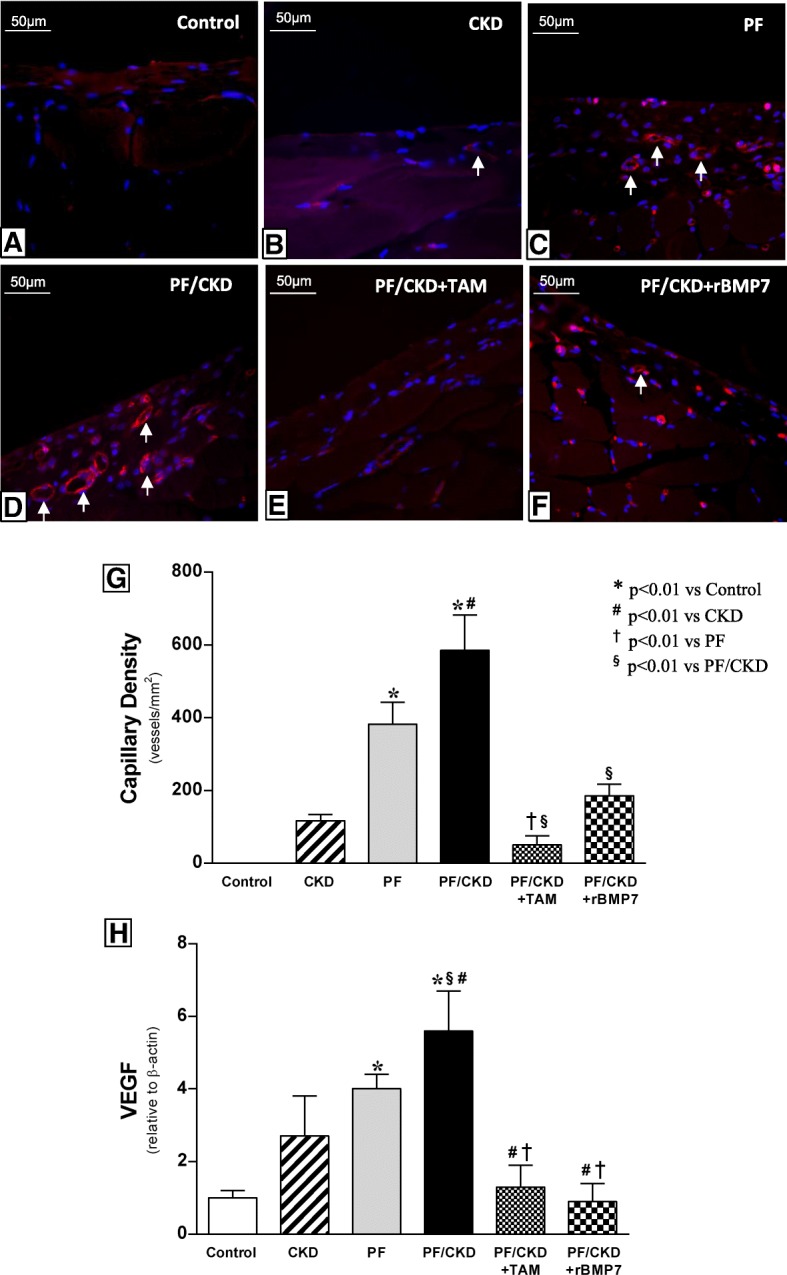
Capillary density and VEGF expression in the peritoneum. Isolectin-B4 positive blood vessels stained in bright red. Nuclei counterstained with DAPI appear in blue (× 400). Almost no vessels were identified in the Control (**a**) and CKD (**b**) groups. Conversely, increased number of capillary vessels indicating neoangiogenesis (white arrows) in the submesothelial zone was observed in the PF (**c**) and CKD/PF (**d**) groups. Tamoxifen (**e**) and rBMP7 (**f**) treatments were effective in reducing the neoangiogenesis. (**g**) Quantitative analysis of the capillary density of all groups at day 30. (**h**) VEGF mRNA levels expression in PM by quantitative real-time PCR (qRT-PCR) were significantly higher in the PF and PF/CKD groups compared with the Control and CKD groups. Both treatments, with TAM or BMP7, significantly reduced VEGF. Overall ANOVA *p* < 0.0001. Post-test *p*-values: *p < 0.01 vs Control; ^#^*p* < 0.01 vs CKD, ^†^*p* < 0.01 vs PF, ^§^*p* < 0.01 vs PF/CKD

The capillary density in the PM analyzed by isolectin B4 expression was significantly increased not only in the PF but especially in the PF/CKD group, as compared with Control and CKD groups, indicating neoangiogenesis (Fig. 2). TAM or rBMP7 treatments inhibited the neovascularization developed in this model (*p* < 0.01 vs PF and CKD/PF). In parallel, the expression of VEGF in PM was significantly higher in the PF group, especially in the PF/CKD group, in relation to the control group. Both TAM and rBMP7 treatments were effective in blocking the expression of VEGF in this setting (*p* < 0.01 vs PF and CKD/PF).

### Tamoxifen and rBMP7 preserved PM function

**Fig. 3:**
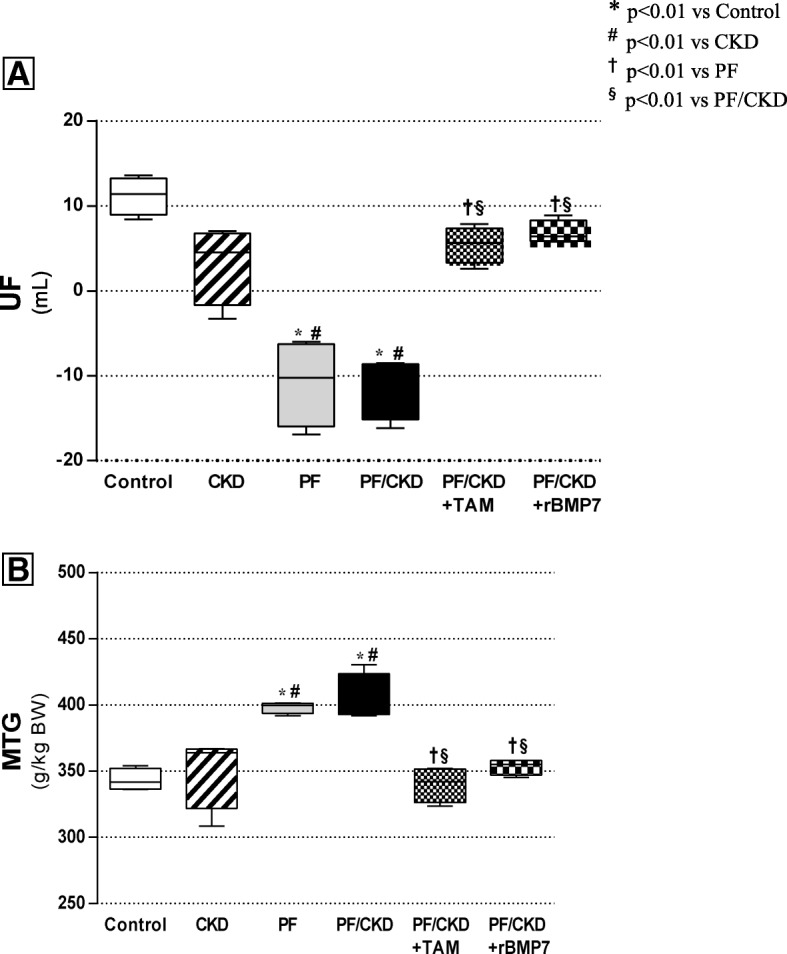
TAM and BMP-7 preserved the ultrafiltration rate (**a**) and reduced the mass transfer of glucose (MTG) (**b**). Overall ANOVA *p* < 0.0001. Post-test p-values: *p < 0.01 vs Control; ^#^*p* < 0.01 vs CKD, ^†^*p* < 0.01 vs PF, ^§^*p* < 0.01 vs PF/CKD

To determine whether the PM morphological preservation induced by TAM or rBMP7 treatment also had an impact on preserving peritoneal function in uremic rats with PF, we analyzed the ultrafiltration (UF) rate and the MTG. Peritoneal function was significantly affected in the PF and PF/CKD groups (Fig. 3). In the PF and CKD/PF groups, a clear and important reduction in the UF rate and an increase in the MTG were observed. Interestingly, TAM and rBMP7 prevented peritoneal function loss by maintaining the UF rate and the MTG at normal levels.

### Tamoxifen and rBMP7 ameliorated fibrogenesis by decreasing the number of myofibroblasts and attenuating the expression of ECM and fibrotic markers in the peritoneum

**Fig. 4:**
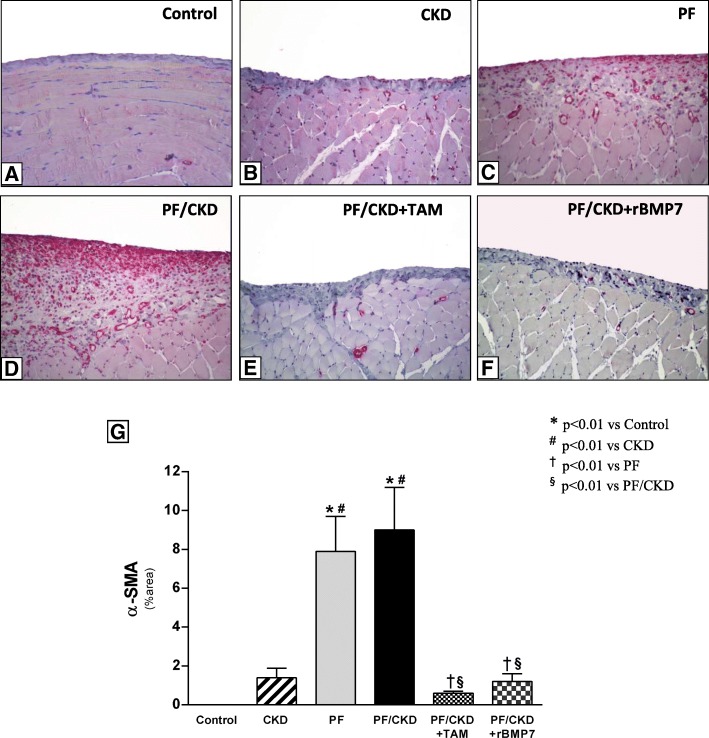
Myofibroblasts were detected in the peritoneal membrane samples of all groups by the expression of α-smooth muscle actin (α-SMA), using immunohistochemistry (× 200). No α-SMA expression was detected in the peritoneal membrane of the Control (**a**) or CKD group (**b**). CG induced PF was associated with a marked increase in α-SMA expression in the PF (**c**) and CKD/PF groups (**d**). Animals with PF and uremia treated with TAM (**e**) or rBMP7 (**f**) exhibited important reduction in the number of myofibroblasts, as demonstrated via quantitative analysis (**g**). Overall ANOVA *p* < 0.0001. Post-test *p*-values: *p < 0.01 vs Control; ^#^*p* < 0.01 vs CKD, ^†^*p* < 0.01 vs PF, ^§^*p* < 0.01 vs PF/CKD

**Fig. 5:**
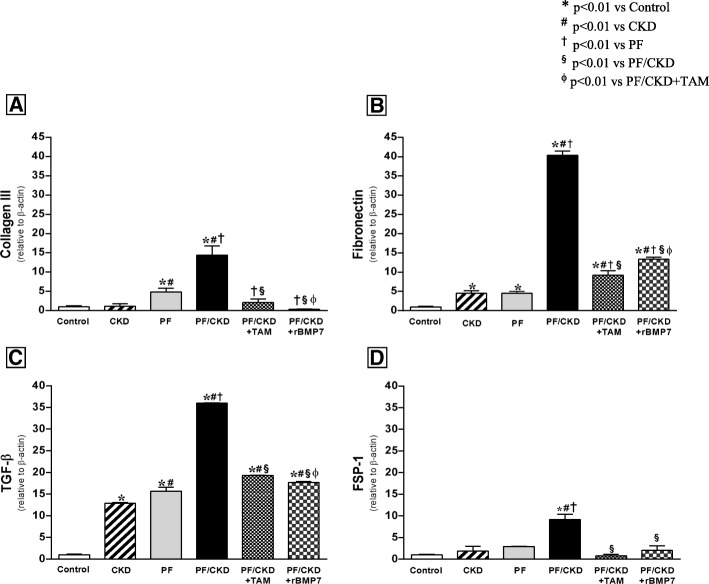
Comparative analysis of collagen III (**a**), fibronectin (**b**), and fibrotic markers in the peritoneal membrane as TGF-β (**c**) and FSP-1 (**d**) of all groups. mRNA levels were measured by quantitative real-time PCR (qRT-PCR). TAM and rBMP7 modulated the mRNA expression of all pro-fibrotic markers. Overall ANOVA *p* < 0.0001. Post-test *p*-values: *p < 0.01 vs Control; ^#^*p* < 0.01 vs CKD, ^†^*p* < 0.01 vs PF, ^§^*p* < 0.01 vs PF/CKD, ^ϕ^*p* < 0.01 vs PF/CKD + TAM

α-SMA, a common marker of myofibroblasts, considered effector cells of fibrogenesis, was strongly expressed in the PF and PF/CKD groups (Fig. 4). Treatment with TAM or rBMP7 substantially reduced the number of myofibroblasts in the PM in this model. The expression of ECM proteins, such as collagen III and fibronectin, as well as other critical molecules involved in the fibrotic process, such as TGF-β and FSP-1, were also investigated by qRT-PCR (Fig. 5). TAM or rBMP7 treatment prevented the increased collagen III and fibronectin expression that occurred in the PF/CKD group. Although TGF-ß expression was markedly increased in the peritoneum of uremic animals with PF, the striking augmented TGF-β expression in the PF/CKD group is noteworthy. More importantly, treatment with TAM or rBMP7 significantly reduced the TGF-β expression as well as FSP-1 expression in PF/CKD animals.

### Tamoxifen and rBMP7 protected against PF, possibly by inhibiting the TGF-β/Smad pathway

**Fig. 6:**
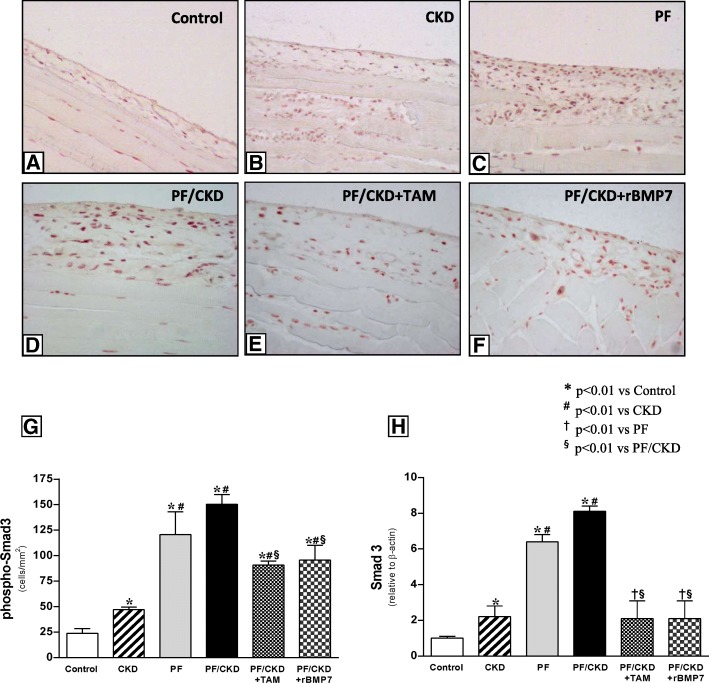
Immunostaining of phospho-Smad3 in the different groups (× 200). Muscle tissue served as a positive control for the immunohistochemistry reaction. Only a few positive cells were noted in the peritoneal membrane of the Control group (**a**). CKD animals exhibited increased cellular expression of phosphorylated Smad3 (**b**). Large numbers of phospho-Smad3-positive cells were detected in the thickened peritoneal membrane of animals with PF (**c**), and in animals with PF and uremia PF (**d**). TAM and rBMP7 significantly blocked Smad3 phosphorylation in the peritoneum (**e** and **f**), as demonstrated via quantitative analysis (**g**). In addition, TAM and rBMP7 significantly prevented Smad3 mRNA expression (**h**). Overall ANOVA *p* < 0.0001. Post-test *p*-values: *p < 0.01 vs Control; ^#^*p* < 0.01 vs CKD, ^†^*p* < 0.01 vs PF, ^§^*p* < 0.01 vs PF/CKD

Smad3, a critical intracellular mediator involved in TGF-β signaling, was evaluated in PM samples (Fig. 6). The peritoneal expression of phospho-Smad3 and Smad3 was significantly higher in the PF and PF/CKD groups than in the Control and CKD groups. However, treatment with TAM or rBMP7 significantly reduced the gene expression and the number of phospho-Smad3^+^ cells in the PM.

**Fig. 7:**
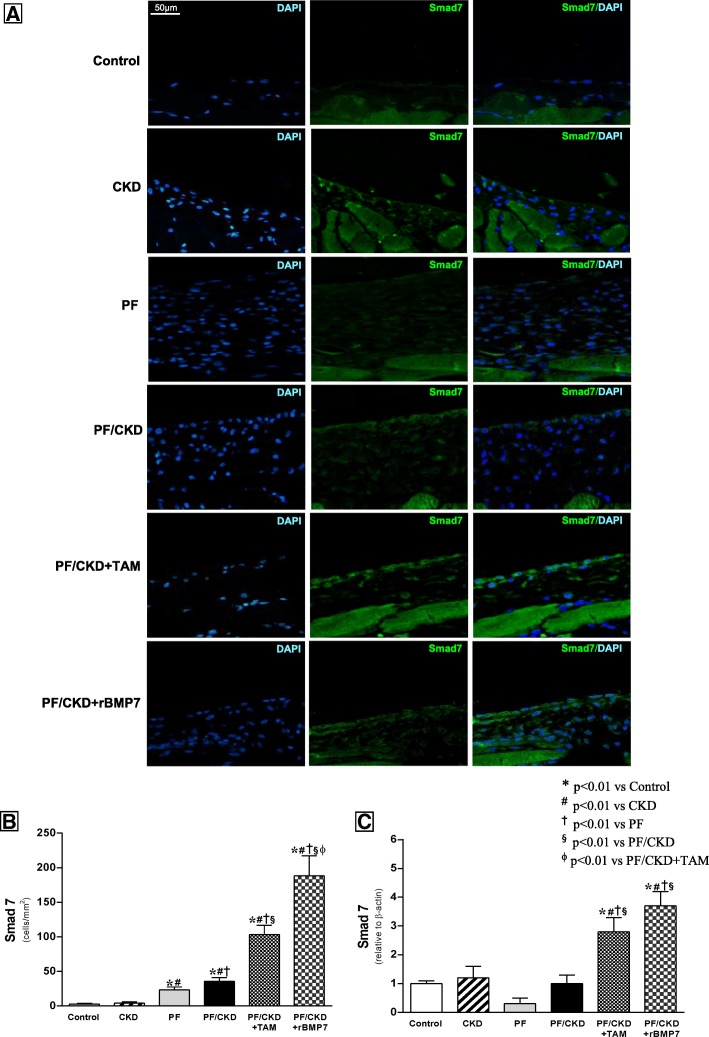
**a**. Immunofluorescence photomicrographs for Smad7 in the peritoneal membrane of the different groups (× 400). Muscle tissue served as a positive control for the immunofluorescence reaction. Only a few positive cells were detected in the Control and CKD groups. Numerous Smad7-positive cells were observed in the PF and CKD/PF groups. TAM and rBMP7 promoted an important increase in the number of Smad7-positive cells in the peritoneal membrane. **b** Quantification analysis demonstrated significantly higher numbers of Smad7-positive cells in the TAM and rBMP7 groups than in all other groups. **c** qRT-PCR confirmed the above immunofluorescence findings, showing markedly increased Smad7 mRNA expression in the peritoneal membrane of the TAM and rBMP7 groups compared to all the other groups. Overall ANOVA *p* < 0.0001. Post-test *p*-values: *p < 0.01 vs Control; ^#^*p* < 0.01 vs CKD, ^†^*p* < 0.01 vs PF, ^§^*p* < 0.01 vs PF/CKD, ^ϕ^*p* < 0.01 vs PF/CKD + TAM

Smad7 expression was also analyzed in PM tissue. We observed low Smad7 expression levels in the CKD, PF and PF/CKD groups. (Fig. 7). In contrast, TAM and rBMP 7 significantly up-regulated Smad7 gene and cellular expression compared with Control, CKD, and PF/CKD groups.

### Inflammation in the PM of uremic rats with PF was prevented by tamoxifen and rBMP7

To elucidate the underlying inflammatory mechanisms involved in the enlargement of PM in this model and evaluate the possible protective effects exerted by TAM and rBMP7 treatments, cellular infiltrate and inflammatory mediators were investigated.

**Fig. 8:**
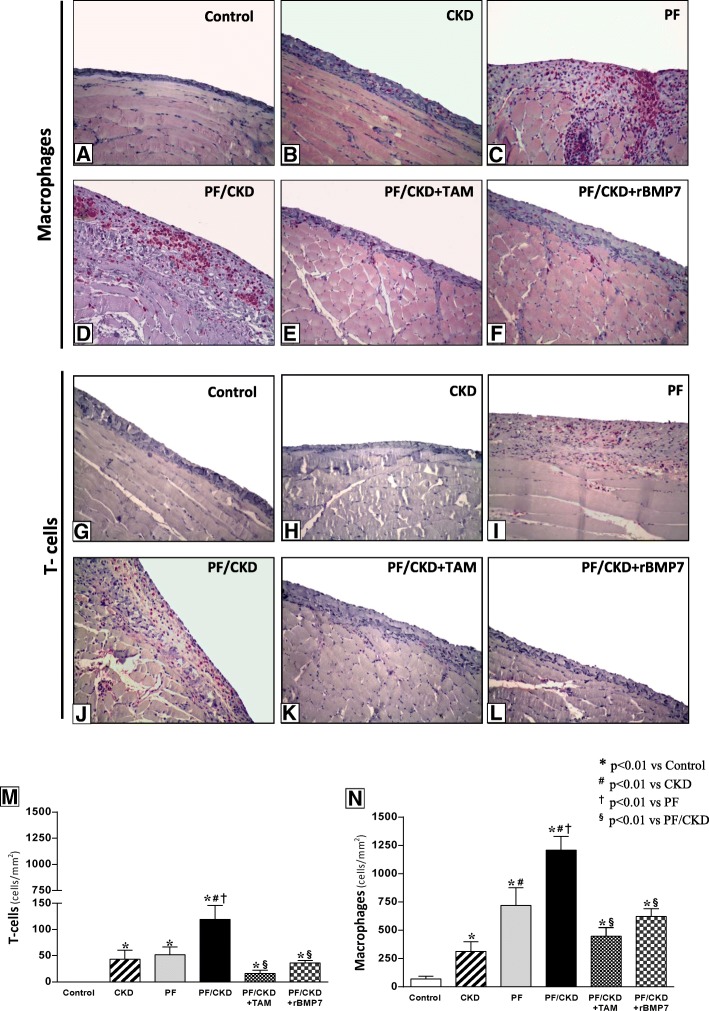
Immunohistochemistry for the detection of macrophages (ED1) and T-cells (CD3) (200x). Only a few ED1+ cells were present in the normal peritoneal membrane (**a**). CKD induced a significant number of macrophages in the peritoneum (**b**). However, the largest number of macrophages was detected in the thickened peritoneal membrane of the PF (**c**) and CKD/PF groups (**d**). TAM (**e**) and rBMP7 (**f**) protected the peritoneal membrane against inflammatory infiltrate comprising macrophages, as shown by quantitative analysis (**m**). A few positive T-cells were detected in the normal peritoneal membrane (**g**). T-cells were also observed in the CKD (**h**) and PF groups (**i**). An important number of T-cells were detected in the CKD/PF group (**j**). TAM (**k**), and rBMP7 (**l**) treatments decreased the number of T-cells in the peritoneal membrane (**n**). Overall ANOVA *p* < 0.0001. Post-test *p*-values: *p < 0.01 vs Control; ^#^*p* < 0.01 vs CKD, ^†^*p* < 0.01 vs PF, ^§^*p* < 0.01 vs PF/CKD

Only a few macrophages (ED1^+^ cells) and T-cells (CD3^+^ cells) were detected in normal PM (Fig. 8). However, the development of CKD and PF promoted significant macrophage and T-cell infiltration, particularly in uremic rats with PF. Treatment with TAM or rBMP7 significantly blocked macrophage infiltration in the PM in this group.

**Fig. 9:**
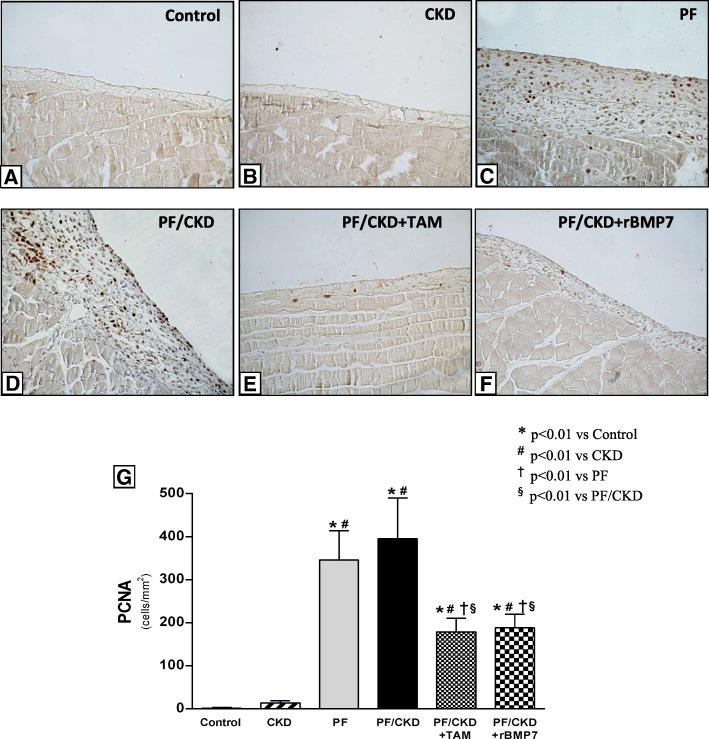
Immunohistochemistry for proliferative cell activity (PCNA) in the peritoneal membrane of the different groups (× 200). No significant proliferative activity was detected in the Control (**a**) or CKD groups (**b**). Peritoneal fibrosis induction promoted increased PCNA expression in the PF (**c**) and CKD/PF (**d**) groups. The groups treated with TAM (**e**) or rBMP7 (**f**) exhibited significantly reduced PCNA expression (**g**). Overall ANOVA *p* < 0.0001. Post-test *p*-values: *p < 0.01 vs Control; ^#^*p* < 0.01 vs CKD, ^†^*p* < 0.01 vs PF, ^§^*p* < 0.01 vs PF/CKD

Additionally, a marked activity of cellular proliferation, measured by PCNA expression, in both the PF and the PF/CKD groups was observed (Fig. 9). In contrast, administration of TAM or rBMP7 effectively prevented the abnormal cellular proliferation.

**Fig. 10:**
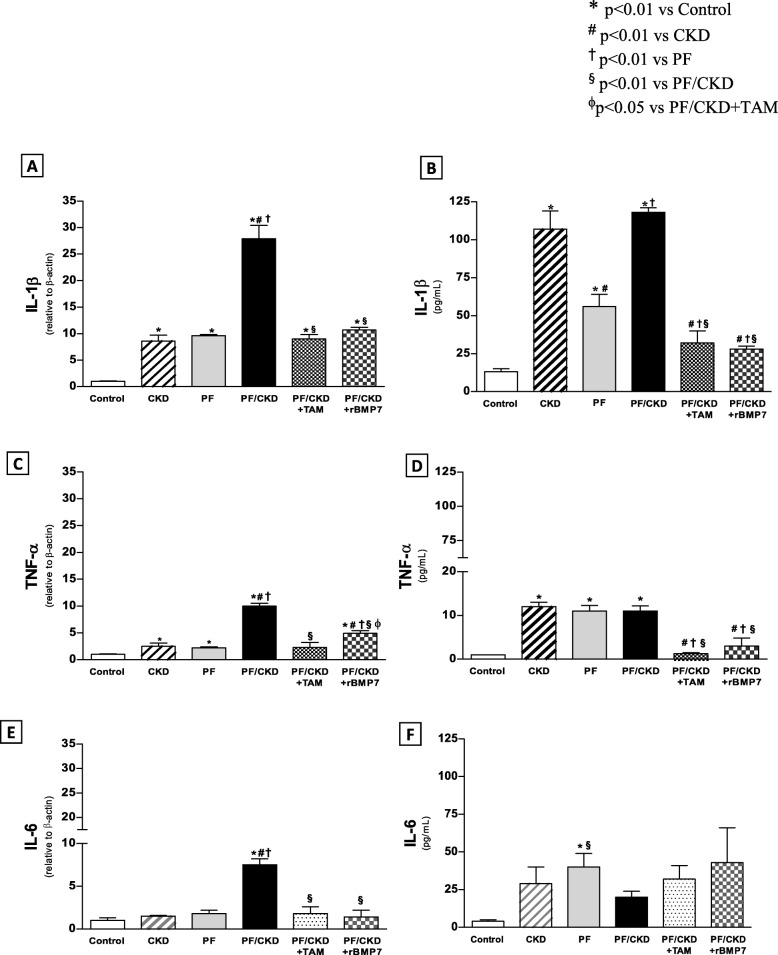
Comparative analysis of inflammatory cytokine expression in the peritoneal membrane of the different groups. mRNA expression of (**a**)  IL1β, (**c**) TNF-α and (**e**) IL6, and protein concentration of (**b**) IL1β, (**d**) TNF-α and (**f**) IL6,  were measured by qRT-PCR and MILLIPLEX MAP, respectively. Overall ANOVA *p* < 0.0001. Post-test *p*-values: *p < 0.01 vs Control; ^#^*p* < 0.01 vs CKD, ^†^*p* < 0.01 vs PF, ^§^*p* < 0.01 vs PF/CKD, ^ϕ^*p* < 0.01 vs PF/CKD + TAM

Cytokine-mediated inflammatory mechanisms in the PM were investigated by measuring TNF-α, IL-1β and IL-6 expression (Fig. 10). IL-1β and TNF-α mRNA expression and protein concentration were significantly higher in the CKD, PF, and CKD/PF groups compared with the Control group. Treatment with TAM or rBMP7 significantly decreased gene expression and protein concentration of these cytokines in the PM. IL-6 mRNA expression was significantly higher in the PF/CKD group than in the Control and PF groups. TAM and rBMP7 prevented IL-6 at mRNA expression, but not at the protein level.

### Treatment of TGF-ß-stimulated peritoneal fibroblasts with tamoxifen or rBMP7 inhibited IL-1ß expression

**Fig. 11:**
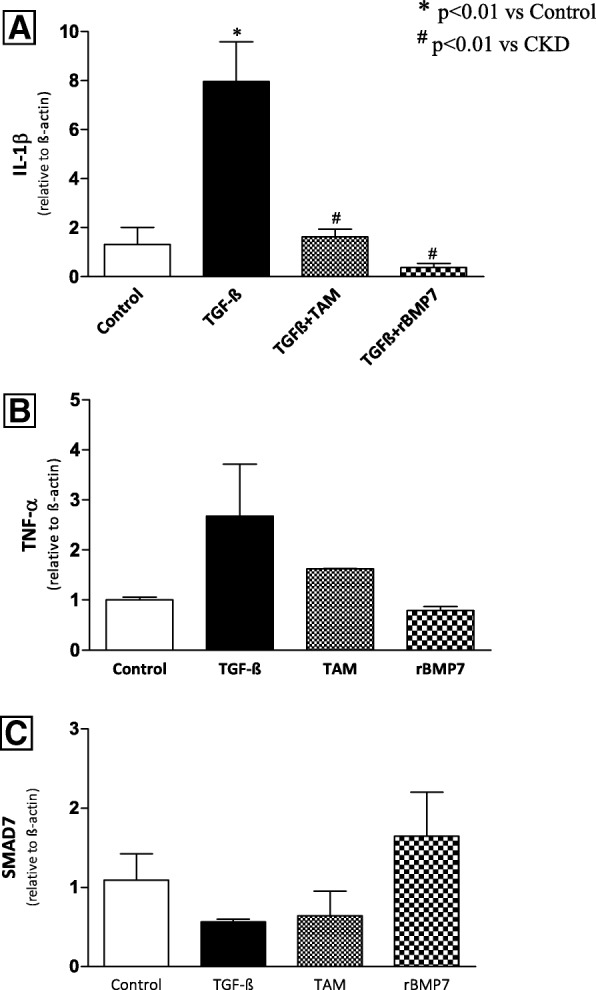
TGF-β stimulation of primary culture peritoneal fibroblasts. Results show mRNA levels measured by quantitative real-time PCR (qRT-PCR) of (**a**) IL1β expression **b**) TNF-α expression, and **c**) Smad7 gene expression. Overall ANOVA *p* < 0.0001. Post-test *p*-values: *p < 0.01 vs Control; ^#^*p* < 0.01 vs CKD

In order to further evaluate the TGF-β-driven inflammatory effects on peritoneal cells, primary culture peritoneal fibroblasts were stimulated with TGF-ß in vitro. Exposure of cells to TGF-ß for 24 h induced a significantly increased expression of IL-1ß (Fig. 11). Exposure of TGF-ß stimulated peritoneal fibroblasts to TAM or rBMP7 reduced the pro-inflammatory cytokine IL-1ß. Similar results, but not statistically significant, were observed for TNF-α expression. Although the results of the TGF-ß stimulation of peritoneal fibroblasts showed a trend towards a decreased Smad7 expression, which was reversed by TAM or rBMP7 treatments, it did not reach statistical significance.

### The anti-inflammatory effects of tamoxifen and rBMP7 in vivo are possibly mediated by IκBα up-regulation

**Fig. 12:**
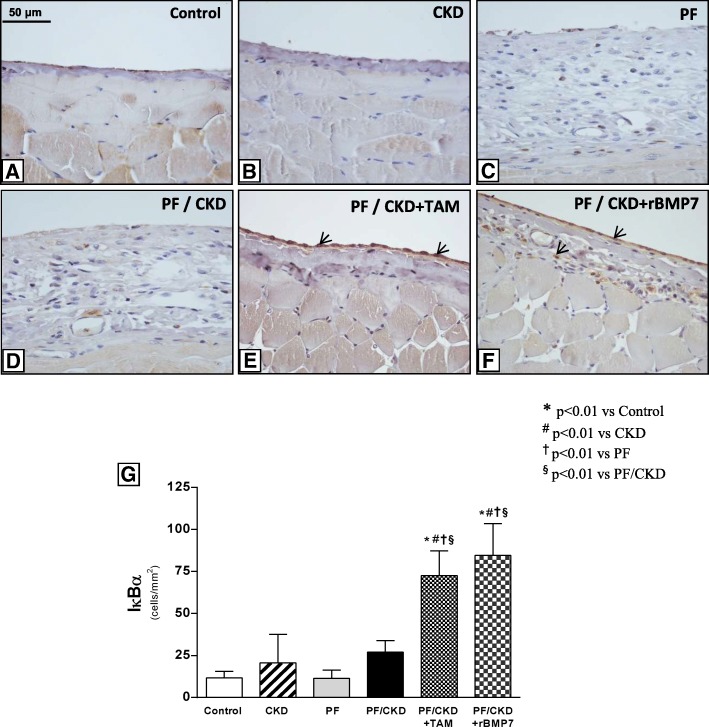
Immunohistochemical photomicrographs for IκB-α detection in the different groups (× 200). Only a few positive cells were observed in the Control and CKD groups (**a** and **b**). Similar expression profiles were observed in the PF and CKD/PF groups (**c** and **d**). TAM and rBMP7 markedly increased IκB-α positivity in the PM (**e**, **f** and **g**). Overall ANOVA *p* < 0.0001. Post-test *p*-values: *p < 0.01 vs Control; ^#^*p* < 0.01 vs CKD, ^†^*p* < 0.01 vs PF, ^§^*p* < 0.01 vs PF/CKD

Given that TAM and rBMP7 upregulated Smad7 expression in the PM, we investigated whether these treatment strategies could also induce the synthesis of the inhibitor of kB-α (IkB-α) in PM, which may exert a key anti-inflammatory role. Immunohistochemistry experiments to detect IκBα in the PM showed a low expression of IκBα in the Control, CKD, PF and PF/CKD groups (Fig. 12). In contrast, treatment with TAM or rBMP7 significantly up-regulated IκBα expression in the PM of uremic rats with PF.

## Discussion

The combo model employed in this study, by combining PF and CKD with uremia, promoted a striking PM thickness accompanied by ECM accumulation, neoangiogenesis, increased cell proliferation, and marked inflammatory cell infiltration in the peritoneum. It is noteworthy that the presence of advanced uremia substantially aggravated the development of PF, a process characterized by significantly increased expression of collagen III, fibronectin, and TGF-ß. Low mortality and low dropout rates represented additional advantages of this model (Mortier et al. 2005).

The major findings of this study were that TAM and rBMP7 exerted protective effects against the fibrogenic process in this combo model of PF with uremia. TAM and rBMP7 treatments were effective in preventing increases in PM thickness in parallel with down-regulation of ECM expression and reduction in the number of myofibroblasts in the peritoneum. Consistent with these results, Loureiro et al. showed that TAM administration significantly diminished peritoneal thickness and inhibited mesothelial-to-mesenchymal transition, but in nonuremic mice with PF (Loureiro et al. 2013). Prevention of PM damage was also demonstrated using rBMP7, being more than two-fold higher than in the PF group. The upregulated expression of TGF- certainly plays a central role in the PF process by promoting increased ECM production, myofibroblast differentiation, and other wide spectrum of biological functions (Margetts et al. 2001; Duan et al. 2014). In fact, induction of TGF-ß expression in the peritoneum through adenovirus-mediated gene transfer has been shown to induce PM thickening, myofibroblastic differentiation and increased ECM production (Margetts et al. 2001; Duan et al. 2014), whereas directly blocking TGF-ß through synthetic peptides protects against PM damage (Loureiro et al. 2011).

The remarkable antifibrotic effects exerted by TAM and rBMP7 in this model of PF with uremia are possibly mediated by TGF-ß inhibition (Loureiro et al. 2011; Dellê et al. 2012), confirming previous studies in other models of PD in the absence of uremia (Loureiro et al. 2011; Loureiro et al. 2013). In parallel to these morphological protective effects, treatment with TAM or rBMP7 also preserved the peritoneal function, by maintaining an adequate peritoneal ultrafiltration capacity and mass transfer of glucose (Nie et al. 2007).

Failure of ultrafiltration capacity observed in the PF and PF with uremia animals is possibly a consequence of neoangiogenesis in the PM, as demonstrated by increased capillary density and upregulation of VEGF (Pecoits-Filho et al. 2002; Stavenuiter et al. 2011). It has been shown that the pro-angiogenic factor VEGF and angiopoietin play a key role in the formation of new vessels in PD (Stavenuiter et al. 2011). The specific role of VEGF was confirmed by the demonstration that neutralizing antibodies to VEGF attenuate the peritoneal fibrotic process (Ada et al. 2015). In the present study, treatment with TAM or rBMP7 was also shown to block VEGF expression, which could explain the recovery of vascular density and amelioration of the peritoneal function. The angiogenic process in the PM upon VEGF up-regulation occurs alongside to the development of PF, possibly by mechanisms involving tissue hypoxia, inflammation and also TGF-β-VEGF interactions (Margetts et al. 2001; Kariya et al. 2018; Patel et al. 2010). Thus, the ability of tamoxifen or rBMP7 in blocking TGF-ß in the PM may have contributed to the recovery of these findings.

Activation of downstream TGF-β/Smad signaling pathway in this experimental setting was confirmed by the markedly increased gene expression of Smad3 and phospho-Smad3 isoforms observed in the PM of animals with PF, particularly in the group of PF with uremia. Smad3 has been recognized as a crucial mediator of TGF-ß biological effects in the fibrogenic process of several renal diseases (Kim et al. 2014; Lan et al. 2003), since its deficiency in Smad3 knockout mice resulted in suppression of the fibrotic response (Sato et al. 2003). The specific role of Smad3 in PF development was also demonstrated by protection of PM damage in Smad3 null mice submitted to long-term high glucose peritoneal solution (Duan et al. 2014) or to PF induced by adenovirus-mediated gene transfer of TGF-β (Patel et al. 2010). These data indicate that Smad3 signaling is an important pathway of the TGF-β induced fibrotic effects.

It is noteworthy that TAM or rBMP7 administration significantly inhibited Smad3 expression and prevented its phosphorylation in the peritoneum of uremic rats with PF. The effects of TAM, a selective ER modulator, are possibly related to its influence on the estrogen-signaling pathway. Binding of TAM to the ER-α prevents Smad3 phosphorylation, halting downstream TGF-ß signaling and synthesis of fibrogenic factors (Matsuda et al. 2001). In agreement with our results, TAM reduced phospho-Smad3 expression in TGF-ß activated NRK-49F renal fibroblasts in a dose-dependent fashion (Kim et al. 2014). Similar findings were described in other models, such as the unilateral ureteral obstruction (UUO)-induced renal fibrosis, the pulmonary fibrosis and the hepatic fibrosis models (Kim et al. 2014; Yang et al. 2013). The exact regulatory mechanisms underlying the effects of rBMP7 on the expression of phospho-Smad3 are still not clear, but are possibly related to the inhibition of SnoN degradation, which is necessary for Smad3 transcription (Luo et al. 2010).

One of the most interesting results of this study was that treatment with TAM or rBMP7 induced remarkable increases in Smad7 expression in the peritoneum of uremic rats with PF. Smad7 has been shown to inhibit TGF-ß signal transduction in vitro *(*Chen et al. 2002*)* and in vivo *(*Lan et al. 2003*;* Hou et al. 2005*)* by blocking Smad2/3 TGF-ß receptor-dependent phosphorylation (Nakao et al. 1997; Benchabane and Wrana 2003). In PF models caused by PD solution, upregulation of Smad7 induced by Smad7 transfection blocked Smad2/3 activation, ameliorated PF and improved peritoneal function (Guo et al. 2007; Nie et al. 2007). In the present study, TAM and rBMP7 treatments induced Smad7 expression in the peritoneum, which in turn blocked Smad3 expression, thereby contributing to a negative modulation of profibrotic genes. Increased expression of Smad7 induced by TAM was also described in the UUO model, leading to TGF-ß suppression and diminished renal tubulointerstitial fibrosis (Kim et al. 2014). The findings of BMP-7 responsive elements in the Smad7 gene may represent a possible explanation for the induction of Smad7 by rBMP7 (Benchabane and Wrana 2003).

The increased expression of Smad7 upon TAM or BMP7 treatments was also associated with attenuation of the inflammatory response in this model. Besides the presence of a marked inflammatory cellular infiltration in the PM, pro-inflammatory cytokines, namely IL-1β, TNF-α, and IL-6, were significantly increased in the peritoneum of the CKD, PF and PF/CKD groups. The higher prevalence of macrophages over T-cells in the PM observed in this model, consistent with the findings in patients on PD (Kitterer et al. 2015), reflects a severe inflammatory process (Lai and Leung 2010). In addition, the association between the degree of inflammation, as determined by inflammatory cell infiltration and up-regulation of pro-inflammatory cytokines, with the severity of PM thickness and ECM overexpression, suggests that inflammation severity may have had a direct impact on PM fibrosis development, particularly in the PF/CKD group. In the clinical setting, the combination of peritoneal inflammation and the uremic environment boosts the risk of fibrosis development and significant loss of peritoneal function (Lai and Leung 2010). Relevant to our findings, other clinical studies have shown that the presence of pro-inflammatory cytokines in the dialysate is associated with worsening ultrafiltration rates in patients on PD (Pecoits-Filho et al. 2002; Ates et al. 2000). It is noteworthy that TAM and rBMP7 administration was effective in suppressing inflammatory cell infiltration and inflammatory cytokines in the PM, as observed in other tissues (Dellê et al. 2012; Sugimoto et al. 2007). Our in vitro experiments, confirmed that peritoneal fibroblasts stimulated with TGF-ß induced IL-1ß expression, which was abrogated by TAM or rBMP7 treatments.

The mechanisms involved in the anti-inflammatory effects of TAM and rBMP7 suppressing peritoneal inflammation are unknown, but it seems reasonable to speculate that they are related to the up-regulated expression of Smad7 induced by these interventional strategies. This assumption is supported by the findings that Smad7 overexpression in different models of renal diseases exhibit significantly inflammation suppression (Ka et al. 2007; Ng et al. 2005). Seminal studies have shown that renal inflammation was markedly suppressed in transgenic mice overexpressing latent TGF-ß in the UUO model, and that these mice also exhibited increased Smad7 expression. The key role of Smad7 in the suppression of inflammatory cytokines, such as TNF-α and IL-1ß, was confirmed by doxycycline-regulated Smad7 in kidney tubular cells (Wang et al. 2005). Smad7has been shown to induce IκB-α expression in a time- and dose-dependent manner. IκB-α binds to the NF-kB p50 and p65 dimers, forming an inactive NF-kB complex that is retained in the cytoplasm, and is not translocated to the nucleus. Therefore, Smad7 can act by- inhibiting NF-kB activation (Wang et al. 2005). The increased IkB-α expression induced by TAM or BMP7 suggests that these pharmacological agents act by increasing Smad7 expression which in turn, leads to an increased IkB-α expression.

## Conclusions

**Fig. 13:**
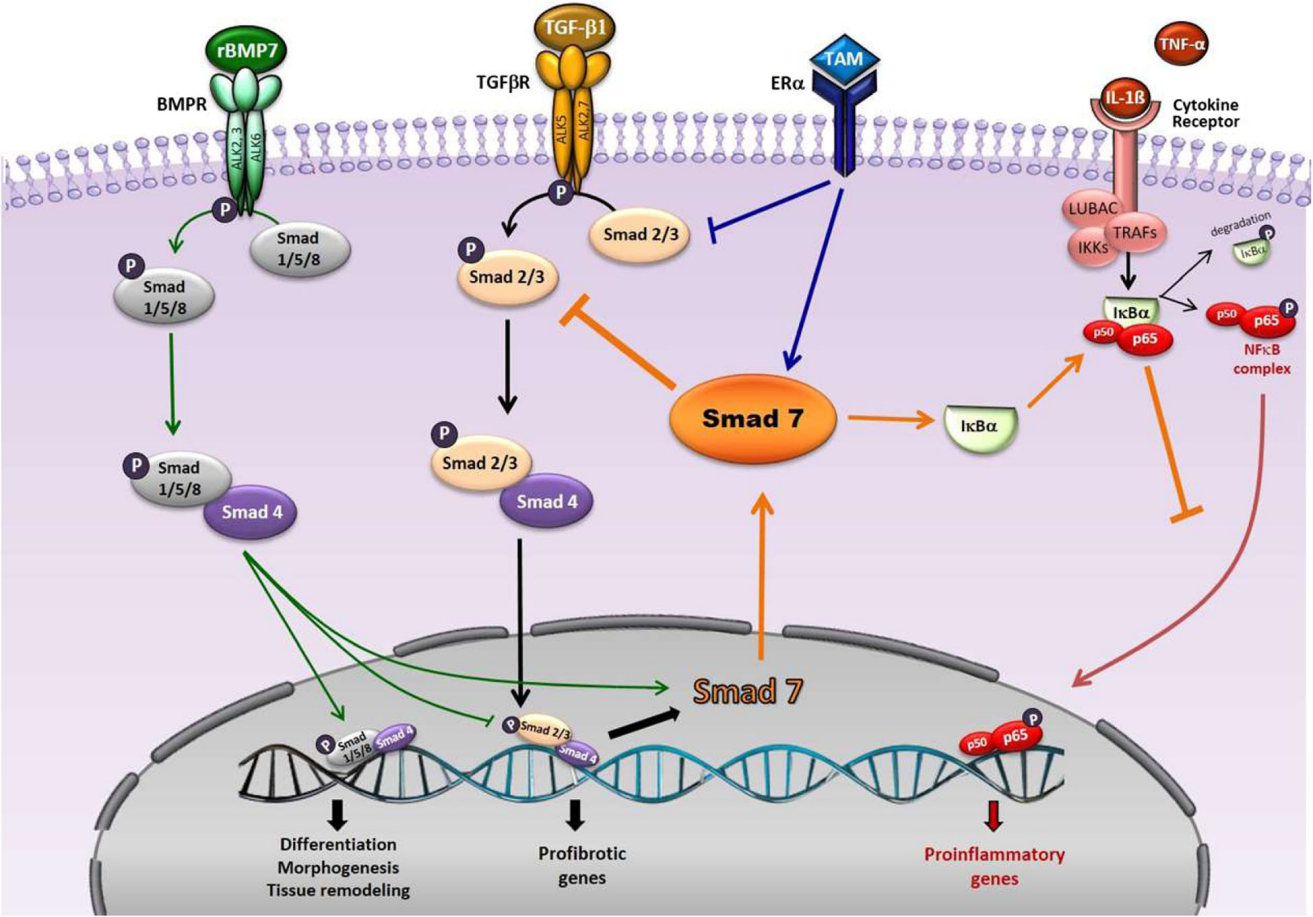
Schematic representation of the TGF-ß/Smad signalling pathway and the possible targets of tamoxifen (TAM) and rBMP7 to prevent PF development. TGF-ß1 binds to the transmembrane serine/threonine kinase cell surface receptor, initiating downstream signal transduction pathways by Smad2 and Smad3 (Smad2/3). phosphorylation. Phosphorylated Smad2/3 forms heteromeric complexes with Smad4, which translocate to the nucleus to regulate the transcription of specific fibrogenic genes, and of Smad7, an intracellular antagonist of TGFβ/Smad signaling. Smad7 interacts with TGF-ß type I receptors, thereby causing receptor ubiquitination and degradation and inhibiting Smad2/3 phosphorylation. Therefore, phosphorylated Smad2/3 interaction with Smad4 is inhibited, providing a negative autoregulation of TGF-ß/Smad signaling. TAM binds to ER-α, which interacts with Smad3, preventing Smad3 phosphorylation. Blocking Smad3 phosphorylation halts downstream TGF-ß signaling and profibrotic factors synthesis. On the other hand, TAM directly increases Smad7 expression, contributing to the negative regulation of TGF-ß signaling. rBMP7 binds to BMP receptors, promoting phosphorylation of Smad1/5/8, which form complexes with Smad4 and translocate to the nucleus to regulate the expression of specific genes. BMP7 inhibits Smad3 DNA binding and induces Smad7 expression to exert negative regulation of TGF-ß pathway. TNF-α and other proinflammatory cytokines bind to their specific cell surface receptors and promote IκBα phosphorylation, which releases both p50/p65 NFκB subunits into the cytoplasm. Phosphorylated IκBα is degraded, and the NF-KB pathway is activated, with subsequent phosphorylation of the p65 subunit, which permits translocation of the subunit to the nucleus to regulate the expression of proinflammatory genes. Smad7 can block TNF-α and other proinflammatory cytokines signaling pathways by up-regulating IκB-α, expression, thereby exerting anti-inflammatory effects

In conclusion, our findings offer exciting insights into the pathophysiology of conditions characterized by local inflammatory responses and tissue fibrosis (Fig. 13). It is noteworthy that TAM and rBMP7 exerted similar effects as anti-fibrotic agents, indicating that blockade of TGF-ß signaling and up-regulation of Smad7 may be effective strategies for preventing the development of PF in the clinical setting.

## Additional file

Additional file 1: **Table S1.** Average body weights and overall mortality rates of all groups. **Table S2.** Urinary volumes of all experimental groups. **Table S3.** Average peritoneal membrane thickness and α-SMA expression in all groups. **Table S4.** Average peritoneal function test values related to ultrafiltration and mass transfer of glucose in all groups. **Table S5.** Analysis of gene expression performed by qRT-PCR for extracellular matrix proteins and profibrotic genes of all groups. **Table S6.** Peritoneal expression of Smad3, phosphorylated Smad3, and Smad7 in all groups. **Table S7.** Quantification of ED1+, CD43+ and PCNA+ cells in the peritoneal membrane in all groups**. Table S8.** Inflammatory cytokine (TNF-α, IL-1β and IL-6) gene and protein expression in the peritoneum. **Table S9.** Quantification of the peritoneal membrane IκBα expression by immunohistochemistry in all groups. **Table S10.** Primer sets used for qRT-PCR. **Figure S1.** Immunofluorescence photomicrographs from phenotypic characterization of cells obtained from primary culture of peritoneal membrane explants, using monoclonal antibodies anti Vimentin (1:200; Sigma), anti-α-SMA, 1:800; Sigma), and anti-desmin (1:200; Sigma), Alexa 488 and Alexa 594 1:200; Life Tchnologies). Cells were positive for vimentin, and negative for α-SMA and desmin, indicating a fibroblast like phenotype. (400x). **Figure S2.** Survival curve during the study period. (DOCX 1124 kb)

## Data Availability

All data generated or analyzed during this study are included in this published article and its additional files.

## Abbreviations

BMP7: Bone morphogenic protein 7
BUN: Blood urea nitrogen
CG: Chlorhexidine gluconate
CKD: Chronic kidney disease
ECM: Extracellular matrix
ER: Estrogen receptor
FSP-1: Fibroblast-specific protein 1
IgG: Immunoglobulin G
IL-1ß: Interleukin 1 beta
IL-6: Interleukin 6
IP: Intraperitoneal
IκB-α: kappa light polypeptide gene enhancer in B-cells inhibitor alpha
PCNA: Proliferating cell nuclear antigen
PD: Peritoneal dialysis
PF: Peritoneal fibrosis
PM: Peritoneal membrane
rBMP7: recombinant BMP7
TAM: Tamoxifen
TGF-ß: Transforming growth factor beta
TNF-α: Tumor necrosis factor alpha
VEGF: Vascular endothelial growth factor
α-SMA: alpha smooth muscle actin
