# Supplementary material for: Tamoxifen and bone morphogenic protein-7 modulate fibrosis and inflammation in the peritoneal fibrosis model developed in uremic rats
Source: Mol Med. 2019 Aug 28;25:41. doi: 10.1186/s10020-019-0110-5 (PMC6712623; doi:10.1186/s10020-019-0110-5)
Supplement: Supplementary file 1 — Table S1. Average body weights and overall mortality rates of all groups. Table S2. Urinary volumes of all experimental groups. Table S3. Average peritoneal membrane thickness and α-SMA expression in all groups. Table S4. Average peritoneal function test values related to ultrafiltration and mass transfer of glucose in all groups. Table S5. Analysis of gene expression performed by qRT-PCR for extracellular matrix proteins and profibrotic genes of all groups. Table S6. Peritoneal expression of Smad3, phosphorylated Smad3, and Smad7 in all groups. Table S7. Quantification of ED1+, CD43+ and PCNA+ cells in the peritoneal membrane in all groups. Table S8. Inflammatory cytokine (TNF-α, IL-1β and IL-6) gene and protein expression in the peritoneum. Table S9. Quantification of the peritoneal membrane IκBα expression by immunohistochemistry in all groups. Table S10. Primer sets used for qRT-PCR. Figure S1. Immunofluorescence photomicrographs from phenotypic characterization of cells obtained from primary culture of peritoneal membrane explants, using monoclonal antibodies anti Vimentin (1:200; Sigma), anti-α-SMA, 1:800; Sigma), and anti-desmin (1:200; Sigma), Alexa 488 and Alexa 594 1:200; Life Tchnologies). Cells were positive for vimentin, and negative for α-SMA and desmin, indicating a fibroblast like phenotype. (400x). Figure S2. Survival curve during the study period. (DOCX 1124 kb) [file 10020_2019_110_MOESM1_ESM.docx]

**Additional file Data legends**

**Table S1.** Average body weights and overall mortality rates of all groups

**Table S2.** Urinary volumes of all experimental groups

**Table S3.** Average peritoneal membrane thickness and α-SMA expression in all groups

**Table S4.** Average peritoneal function test values related to ultrafiltration and mass transfer of glucose in all groups

**Table S5.** Analysis of gene expression performed by qRT-PCR for extracellular matrix proteins and profibrotic genes of all groups

**Table S6.** Peritoneal expression of Smad3, phosphorylated Smad3, and Smad7 in all groups

**Table S7.** Quantification of ED1+, CD43+ and PCNA+ cells in the peritoneal membrane in all groups

**Table S8.** Inflammatory cytokine (TNF-α, IL-1β and IL-6) gene and protein expression in the peritoneum

**Table S9.** Quantification of the peritoneal membrane IκBα expression by immunohistochemistry in all groups

**Table S10.** Primer sets used for qRT-PCR

**Figure S1.** Immunofluorescence photomicrographs from phenotypic characterization of cells obtained from primary culture of peritoneal membrane explants, using monoclonal antibodies anti Vimentin (1:200; Sigma), anti-α-SMA, 1:800; Sigma), and anti-desmin (1:200; Sigma), Alexa 488 and Alexa 594 1:200; Life Tchnologies). Cells were positive for vimentin, and negative for α-SMA and desmin, indicating a fibroblast like phenotype. (400x).

**Figure S2.** Survival curve during the study period

.

**Table S1.** Average body weights and overall mortality rates of all groups

|  | **day 0**  (g) | **day 15**  (g) | **day 30**  (g) | **Δ body weight between day 0 and day 30**  (g) | **% of**  **Δ body weight**  (g) | **Overall mortality**  (n) |
| --- | --- | --- | --- | --- | --- | --- |
| **Control** (n=9) | 334 ± 4 | 362 ± 4 | 429 ± 4 | 94 ± 6 | 28% | 0 |
| **CKD** (n=13) | 323 ± 5 | 296 ± 5 | 244 ± 8*^†^ | -79 ± 8*^†^ | -24% | 4 |
| **PF** (n=9) | 315 ± 6 | 364 ± 6 | 371 ± 7 | 56 ± 8* | 18% | 0 |
| **PF/CKD** (n=17) | 312 ± 5 | 266 ± 4*^†^ | 240 ± 10*^†^ | -72 ± 9*^†^ | -23% | 6 |
| **PF/CKD+TAM** (n=10) | 320 ± 9 | 263 ± 8*^†^**^#^** | 227 ± 9*^†^ | -93 ± 2*^†^ | -29% | 0 |
| **PF/CKD+rBMP7** (n=10) | 312 ± 4 | 253 ± 5*^†^**^#^** | 262 ± 6*^†^φ | -51 ± 2***^†^**φ | -16% | 0 |

Data are expressed as mean ± SEM

* p<0.01 vs Control

**#** p<0.01 vs CKD

**†** p<0.01vs PF

**§** p<0.01 vs PF/CKD

φ p<0.05 vs PF/CKD+TAM

**Table S2.** Urinary volumes of all experimental groups

|  | **day 0**  (mL/24h) | **day 15**  (mL/24h) | **day 30**  (mL/24h) |
| --- | --- | --- | --- |
| **Control** | 13 ± 1 | 14 ± 1 | 11 ± 1 |
| **CKD** | 13 ± 1 | 75 ± 3^*^**^†^** | 84 ± 1^*^**^†^** |
| **PF** | 12 ± 1 | 14 ± 1 | 11 ± 1 |
| **PF/CKD** | 15 ± 3 | 77 ± 3^*^**^†^** | 83 ± 3^*^**^†^** |
| **PF/CKD+TAM** | 15 ± 1 | 84 ± 1^*^**^†^** | 79 ± 3^*^**^†^** |
| **PF/CKD+rBMP7** | 16 ± 2 | 80 ± 3^*^**^†^** | 83 ± 2^*^**^†^** |

Data are expressed as mean ± SEM

* p<0.01 vs Control

**†** p<0.01vs PF

**Table S3.** Average peritoneal membrane thickness and α-SMA expression in all groups

|  | **PM Thickness**  (µm) | **α-SMA**  ( % area) |
| --- | --- | --- |
| **Control** | 35.4± 2.8 | 0 ± 0 |
| **CKD** | 25.6 ± 3.1**^†^** | 1.6 ± 0.5**^†^** |
| **PF** | 125.0± 3.1* | 7.9 ± 1.8* |
| **PF/CKD** | 132.9 ± 22.2***^#^** | 9.0 ± 2.2***^#^** |
| **PF/CKD+TAM** | 43.5 ± 3.0 **^†§^** | 0.6 ± 0.1**^†§^** |
| **PF/CKD+rBMP7** | 57.6 ± 8.0**^†§^** | 1.2 ± 0.4**^†§^** |

Data are expressed as mean ± SEM

* p<0.01 vs Control

**#** p<0.01 vs CKD

**†** p<0.01vs PF

**§** p<0.01 vs PF/CKD

**Table S4.** Average peritoneal function test values related to ultrafiltration and mass transfer of glucose in all groups

|  | **UF**  (mL) | **MTG**  (g/Kg of body weight) |
| --- | --- | --- |
| **Control** | 3.3 ± 3.6 | 343 ± 4 |
| **CKD** | 3.1 ± 2.3† | 350 ± 14† |
| **PF** | -10.8 ± 2.5* | 398 ± 2* |
| **PF/CKD** | -11.4 ± 1.7*# | 405 ± 8*# |
| **PF/CKD+TAM** | 5.4 ± 1.1†^§^ | 340 ± 6†^§^ |
| **PF/CKD+rBMP7** | 6.9 ± 0.7†^§^ | 353 ± 3†^§^ |

Data are expressed as mean ± SEM

* p<0.01 vs Control

**#** p<0.01 vs CKD

**†** p<0.01vs PF

**§** p<0.01 vs PF/CKD

**Table S5.** Analysis of gene expression performed by qRT-PCR for extracellular matrix proteins and profibrotic genes of all groups

|  | **Collagen III**  mRNA | **Fibronectin**  mRNA | **FSP-1**  mRNA | **TGF-β**  mRNA |
| --- | --- | --- | --- | --- |
| **Control** | 1.0 ± 0.2 | 1.0 ± 0.1 | 1.0 ± 0.1 | 1.0 ± 0.1 |
| **CKD** | 1.1 ± 0.7**†** | 4.5 ± 0.7* | 1.9 ± 1.1 | 12.9 ± 0.2***†** |
| **PF** | 4.8 ± 1* | 4.5 ± 0.5* | 1.9 ± 0.1 | 15.7 ± 0.9* |
| **PF/CKD** | 14.4 ± 2.4*†# | 40.4 ± 1.1***†#** | 9.2 ± 1.2***†#** | 36.0 ± 0.1***†#** |
| **PF/CKD+TAM** | 2.1 ± 0.9**†^§^** | 9.2 ± 1.2***†#^§^** | 0.8 ± 0.3**^§^** | 19.3 ± 0.1***#^§^** |
| **PF/CKD+rBMP7** | 0.3 ± 0.1**^†§^**^φ^ | 13.4 ± 0.5*^†#§φ^ | 2.1 ± 1.0**^§^** | 17.7 ± 0.2*^#§φ^ |

Data are expressed as mean ± SEM

* p<0.01 vs Control

**#** p<0.01 vs CKD

**^†^** p<0.01vs PF

**^§^** p<0.01 vs PF/CKD

^φ^ p<0.05 vs PF/CKD+TAM

**Data 6.** Peritoneal expression of Smad3, phosphorylated Smad3, and Smad7 in all groups

|  | **Smad3**  (cells/mm^2^) | **phospho-Smad3**  (cells/mm^2^) | **Smad7**  (cells/mm^2^) |
| --- | --- | --- | --- |
| **Control** | 0 | 23.9 ± 4.5 | 2.6 ± 1.1 |
| **CKD** | 1.16 ± 1.16^*†^ | 46.7 ± 2.6^*^ | 4.2 ± 1.8 |
| **PF** | 173.2 ± 53.8^*#^ | 120.8 ± 22.4^*#^ | 23.2 ± 4.3^*#^ |
| **PF/CKD** | 354.8 ± 37.8^*#†§^φ | 150.4 ± 9.6^*#^ | 35.6 ± 5.5^*#†^ |
| **PF/CKD+TAM** | 109.4 ± 15.6^*#§^ | 90.7 ± 4.1^*#§^ | 103.3 ± 13.5^*#†§^ |
| **PF/CKD+rBMP7** | 101.0 ± 25.3^*#§^ | 95.7 ± 14.4^*#§^ | 188.4 ± 29.3^*#†§^φ |

Data are expressed as mean ± SEM

* p<0.01 vs Control

**#** p<0.01 vs CKD

**^†^** p<0.01vs PF

**^§^** p<0.01 vs PF/CKD

φ p<0.05 vs PF/CKD+TAM

**Table S7.** Quantification of ED1+, CD43+, and PCNA+ cells in the peritoneal membrane in all groups

|  | **ED1^+^**  (cells/mm^2^) | **CD43^+^**  (cells/mm^2^) | **PCNA**  (cells/mm^2^) |
| --- | --- | --- | --- |
| **Control** | 68.0 ± 25.1 | 0 ± 0 | 2 ± 1 |
| **CKD** | 325.4 ± 91.2***^†^** | 49.4 ± 17.1* | 15 ± 5**^†^** |
| **PF** | 718.6 ± 157.0* | 52.0 ± 15.4* | 346 ± 68* |
| **PF/CKD** | 1208 ± 123***^†#^** | 119.4 ± 27.0***^†#^** | 395 ± 95***^#^** |
| **PF/CKD+TAM** | 448.2 ± 74***^§^** | 16.4 ± 6.1***^§^** | 179 ± 32***^†#§^** |
| **PF/CKD+rBMP7** | 620.7 ± 71.5***^§^** | 36.0 ± 5.2***^§^**^φ^ | 188 ± 32***^†#§^** |

Data are expressed as mean ± SEM

* p<0.01 vs Control

**#** p<0.01 vs CKD

**†** p<0.01vs PF

**§** p<0.01 vs PF/CKD

φ p<0.05 vs PF/CKD+TAM

**Table S8.** Inflammatory cytokine (TNF-α, IL-1β and IL-6) gene and protein expression in the peritoneum

|  | **TNF-α** | | |  | **IL-1β** | | | | |  | **IL-6** | | | |
| --- | --- | --- | --- | --- | --- | --- | --- | --- | --- | --- | --- | --- | --- | --- |
|  | mRNA | pg/ml | |  | mRNA | | | pg/ml | |  | mRNA | | pg/ml | |
| **Control** | 1.0 ± 0.1 | | 1.0 ± 0 | | |  | 1.0 ± 0.1 | | 13.0 ± 2.0 | |  | 1.0 ± 0.3 | | 4.0 ± 1.0 |
| **CKD** | 2.5 ± 0.6* | | 12.0 ± 1.0* | | |  | 8.6 ± 1.1* | | 107.0 ± 12.0***†** | |  | 1.5 ± 0.1 | | 29.0 ± 11.0***§** |
| **PF** | 2.2 ± 0.2* | | 11.0 ± 1.3* | | |  | 9.6 ± 0.2* | | 56.0 ± 8.0* | |  | 1.8 ± 0.4 | | 40.0 ± 9.0***§** |
| **PF/CKD** | 10.0 ± 0.5***†#** | | 11.0 ± 1.2* | | |  | 28.0 ± 2.5***†#** | | 118.0 ± 3.0***†** | |  | 7.5 ± 0.7***†#** | | 20.0 ± 4.0 |
| **PF/CKD+TAM** | 2.3 ± 0.9**§** | | 1.3 ± 0.2 **†#§** | | |  | 9.0 ± 0.8***§** | | 32.0 ± 8.0 **†#§** | |  | 1.8 ± 0.8**§** | | 31.0 ± 9.0 |
| **PF/CKD+rBMP7** | 4.9 ± 0.5***†^#§^**^φ^ | | 3.0 ± 1.8 **†#§** | | |  | 10.7 ± 0.5***§** | | 28.0 ± 2.0**†#§** | |  | 1.4 ± 0.8**§** | | 43.0 ± 23.0 |

Data are expressed as mean ± SEM

* p<0.01 vs Control

**#** p<0.01 vs CKD

**†** p<0.01vs PF

**§** p<0.01 vs PF/CKD

φ p<0.05 vs PF/CKD+TAM

**Table S9.** Quantification of the peritoneal membrane IκBα expression by immunohistochemistry in all groups

|  | **Iκβ-α** (cells/mm^2^) |
| --- | --- |
| **Control** | 11.6 ± 4.1 |
| **CKD** | 20.6 ± 17.1 |
| **PF** | 11.4 ± 4.9 |
| **PF/CKD** | 27.0 ± 6.9 |
| **PF/CKD+TAM** | 72.6 ± 14.6***†#§** |
| **PF/CKD+rBMP7** | 84.5 ± 19.0***†#§** |

Data are expressed as mean ± SEM

***** p<0.05vs Control

**#** p<0.05 vs CKD

**†** p<0.05 vs PF

**§** p<0.05 vs PF/CKD

**Table S10.** Primer sets used for qRT-PCR

|  | Primer sequence 5’🡪3’ |
| --- | --- |
| IL-1β | Forward CCTTGTGCAAGTGTCTGAAGCAGC  Reverse GCCACAGCTTCTCCACAGCCA |
| TNF-α | Forward ATCTGAGGGCTCGCCCGGT’  Reverse CAATGGCAGCACCGCCACCA |
| IL-6 | Forward CCGGAGAGGAGACTTCACAGAGGA  Reverse AGCCTCCGACTTGTGAAGTGGTATA |
| TGF-ß | Forward CAACCCGGGTGCTTCCGCAT  Reverse TGCTCCACCTTGGGCTTGCG |
| FSP-1 | Forward GGCAACGAGGGTGACAAGTT  Reverse CCCTGGTCAGTAGTCCCTTGA |
| Collagen type III | Forward ATCTGAGGGCTCGCCCGGT  Reverse CAATGGCAGCACCGCCACCA |
| Fibronectin | Forward TGACCCAGACTTACGGTGGCA  Reverse GGAGTAGAAGGTCCTACCGTTGTAGTG |
| Smad3 | Forward TCAACGGAACTTGGGAATGAG  Reverse TCACCTCGATCTTGACCTTTTGT |
| Smad7 | Forward CCTGGCCGGTGTAAATGTCT  Reverse CGGGATCCCTTGGAAAGG |
| β-actin | Forward AGGAGTACGATGAGTCCGGCCC  Reverse GTAGTGCGGAGCTCTCCTTCA |

**Figure S1.** Immunofluorescence photomicrographs from phenotypic characterization of cells obtained from primary culture of peritoneal membrane explants, using monoclonal antibodies anti Vimentin (1:200; Sigma), anti-α-SMA, 1:800; Sigma), and anti-desmin (1:200; Sigma), Alexa 488 and Alexa 594 1:200; Life Tchnologies). Cells were positive for vimentin, and negative for α-SMA and desmin, indicating a fibroblast like phenotype. (400x)


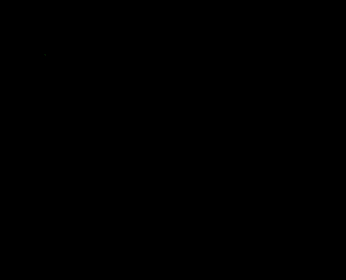


**α-SMA**


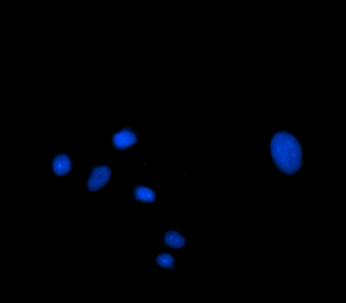


**DAPI**

50µm


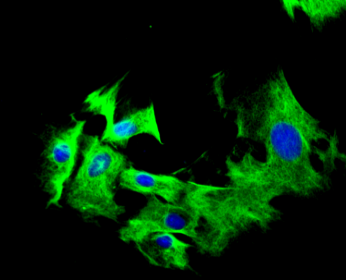


**Vimentin**/**DAPI**

50µm


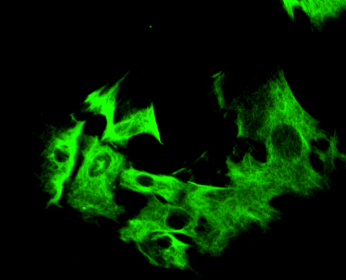


**Vimentin**

50µm


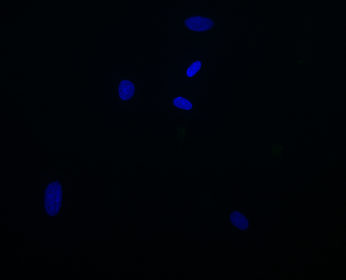


**α-SMA**/**DAPI**


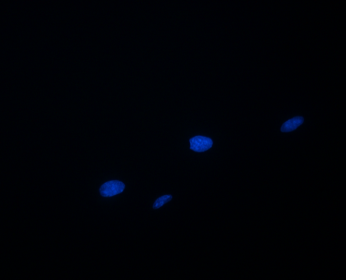


**DAPI**


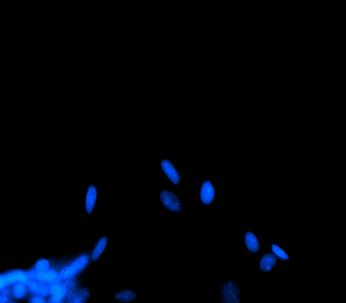


**DAPI**


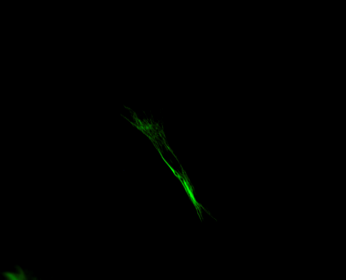


**Desmin**


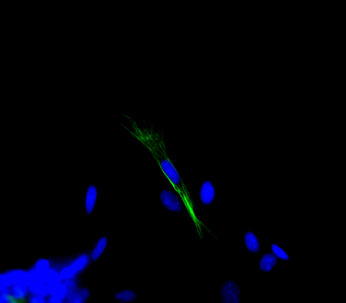


**Desmin**/**DAPI**

**Vimentin**

**α-SMA**

**Desmin**

**Figure S2.** Survival curve during the period of study.

Data are expressed as mean ± SEM

***** p<0.05vs Control

**#** p<0.05 vs CKD

**†** p<0.05 vs PF

**§** p<0.05 vs PF/CKD
